# Insecticide-treated screening of windows for household protection against insecticide-resistant *Anopheles gambiae* sensu lato in Côte d’Ivoire: a semi-field trial

**DOI:** 10.1186/s13071-025-07194-z

**Published:** 2025-12-30

**Authors:** Marius Gonse Zoh, Antoine Barreaux, Edouard Dangbenon, Alphonsine Amanan Koffi, Ludovic Phamien Ahoua Alou, Soromane Camara, N’Guessan Brou, Serge Yao Koffi, Matthew Brian Thomas, Raphael N’Guessan

**Affiliations:** 1 Centre d’Entomologie Médicale et Vétérinaire, Université Alassane Ouattara (CEMV-UAO), Bouaké, Côte d’Ivoire; 2 Vector Control Product Evaluation Centre (VCPEC-IPR/INSP), Bouaké, Côte d’Ivoire; 3 Institut Pierre Richet (IPR)/Institut National de Santé Publique (INSP), Bouaké, Côte d’Ivoire; 4 Cirad, UMR INTERTRYP, Nairobi, Kenya; 5 INTERTRYP, Univ Montpellier, Cirad, Montpellier, IRD France; 6 Animal Health Theme, ICIPE, Nairobi, Kenya; 7 Department of Entomology and Nematology, University of Florida, Gainesville, USA; 8 Department of Biology, University of York, York, UK; 9 London School of Hygiene and Tropical Medicine, London, UK

**Keywords:** Vector control, House modification, Screening, *Anopheles gambiae*, Malaria

## Abstract

**Background:**

Despite the significant impact of long-lasting insecticidal nets (LLINs) and indoor residual spraying (IRS) on malaria, 597,000 deaths from malaria were still recorded in 2023. Additional measures are clearly needed to complement current tools. We assessed the efficacy of insecticide-treated window screens (a piece of PermaNet 3.0 containing deltamethrin + piperonyl butoxide) versus untreated window screening and eave tube inserts treated with β-cyfluthrin as household interventions against *Anopheles gambiae* sensu lato*.*

**Method:**

Between August and October 2022, five experimental houses in Kolongonouan village, central Côte d’Ivoire, were used under semi-field conditions to evaluate the following interventions: (i) standard control house with small openings in the eaves and no window screens (SCH); (ii) insecticide-treated screening (ITS) of windows with small openings in the eaves (ITS op); (iii) ITS of windows with eaves blocked to prevent mosquito entry via eaves (ITS blq); (iv) insecticide treatment applied to the eaves using In2Care eave tubes, without window screening (ET); and (v) untreated window screening together with insecticide-treated eave tubes (SET). The efficacy of these treatments on the indoor and outdoor density of naturally recruiting *Anopheles gambiae* mosquitoes was assessed by human landing catches from 6:00 pm to 8:00 am. The impact of these treatments on mosquito mortality and blood-feeding rates was further assessed using release–recapture experiments, in which large enclosures were built around individual houses and approximately 100 non-blood-fed female *An. gambiae* were released each night over 15 nights. In addition, for the ITS blq treatment, the importance of physical integrity was assessed by evaluating the effect of damaging the screening with two or four 4 cm × 4 cm holes in each screened window.

**Results:**

Insecticide-treated window screening plus eave blocking (ITS blq) significantly reduced by 31.8% the number of *An. gambiae* entering houses overnight relative to standard control houses (SCH) (*P* < 0.001). The use of untreated screening + eave tubes (SET) induced a similar reduction (27.0%, *P* < 0.001). The house entry reduction magnitude for eave tubes alone (ET) was lower (23.5%), though it remained significant relative to the control (*P* < 0.001). The impact of the ITS op (insecticide-treated window screening with open eaves) was lower and nonsignificant (18.1%; *P* = 0.4019). There were no significant differences in house entry rates between ET, ITS blq, and ITS op (*P* > 0.05). The peak in mosquito abundance within houses occurred between 11:00 pm and 3:00 am. There were no significant differences between treatments in the numbers of mosquitoes collected outdoors. In the release–recapture experiments, all treatments significantly reduced the blood-feeding rate and increased the mortality of *An. gambiae*, with again a similar impact for ITS blq and SET. Damaging the ITS with two holes still led to a significant reduction in blood-feeding rate, but protection was lost with four holes. Mortality rate also declined with increasing levels of damage, although it remained significantly higher than that with the untreated window screening.

**Conclusions:**

This study demonstrated that the combination (insecticide-treated window screening and blocking of eave access) reduced mosquito entry, increased mortality, and lowered blood-feeding rates of *An. gambiae* to levels comparable to insecticide-treated eave tubes combined with untreated window screening (SET). A previous cluster randomized controlled trial of SET in this area demonstrated a significant reduction in malaria incidence and prevalence. Insecticide-treated window screens could therefore yield a similar epidemiological impact while potentially being simpler and less expensive to implement. Larger-scale epidemiological trials involving communities are needed to test this assumption and further optimize the approach.

**Graphical abstract:**

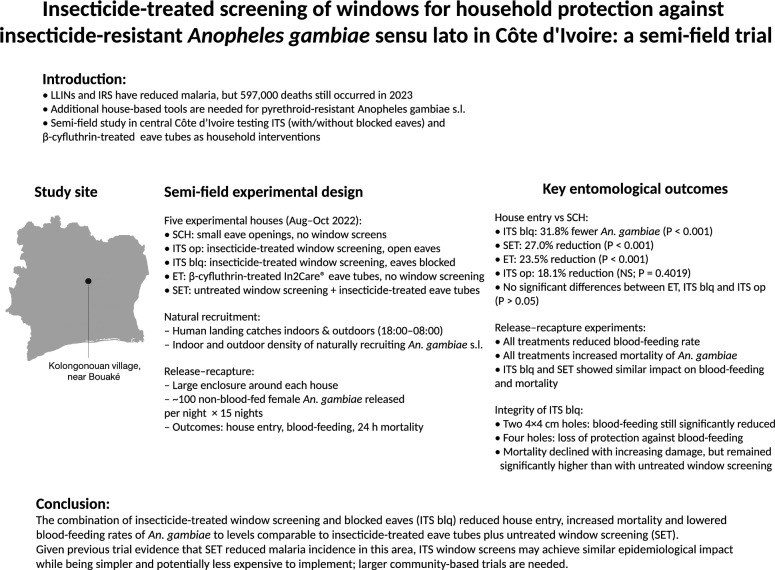

**Supplementary Information:**

The online version contains supplementary material available at 10.1186/s13071-025-07194-z.

## Background

Malaria burden has declined significantly in Sub-Saharan Africa over the past 10–15 years. This reduction is mainly attributed to the large-scale use of insecticide-based interventions, such as indoor residual spraying (IRS) and insecticide-treated nets (ITNs) [1]. Between 2004 and 2019, approximately 1.9 billion ITNs were distributed across the region [1]. However, despite these efforts, malaria elimination has not been achieved in endemic countries, partly attributable to increasing insecticide resistance in malaria vectors [2].

In rural areas of Africa, eaves of houses are a major entry point for malaria vectors, facilitating human exposure to mosquito bites within dwellings and thereby transmission of malaria [3]. To address this, structural innovations have been developed to complement malaria control efforts. One is the eaves tube (ET) technology, developed by IN2CARE, which combines home improvement and vector control. Eave tubes consist of plastic tubes installed in the eaves of houses, fitted with a disc containing electrostatically charged inserts that is treated with insecticide powder [4]. At eave level, these treated inserts target mosquitoes attempting to enter the house, killing them on contact, thereby reducing vector densities indoors [5]. Field trials in Africa demonstrated that eave tubes in combination with screening of windows reduced mosquito densities and malaria transmission when implemented on a large scale [6, 7]. Eave baffles, another intervention that involves installing physical barriers made of either fine mesh, treated fabric, or a metal sheet along the eaves to block mosquito entry, can also be treated with insecticides to boost their effectiveness [8–10]. Despite the efficacy of these interventions against malaria vectors, they can have significant limitations during their use. For example, the efficacy of eave tubes depends on proper installation and regular maintenance. Any gaps, poor wall fit, or deterioration of the insecticide-treated inserts can create entry points that allow mosquitoes to bypass the system, thereby reducing its protective benefit [5]. In addition, eave tubes alone may not provide complete protection if other entry points, such as windows and doors, are left unprotected [4]. Eave baffles, although effective at blocking mosquito entry through open eaves, can obstruct ventilation and require regular maintenance to maintain their efficacy as insecticide treatments degrade over time [10]. In all cases, the absence of window screening or complete sealing of the house significantly reduces the effectiveness of these interventions, underscoring the need for integrated strategies that address all potential mosquito entry points [11]. Moreover, although eave tubes and window screening can be cost-effective [12], the absolute cost per house currently represents a major barrier to large-scale implementation.

Insecticide-treated screening (ITS) has been established as a cost-effective and efficient method of malaria vector control and has demonstrated significant potential in reducing mosquito populations and disease transmission [12]. In Tanzania, insecticide-treated window screens combined with eave baffles reduced *Anopheles* entry and increased mortality [8]. The aim of this study was to assess the impact of the ITS of windows on the behavior of insecticide-resistant *An. gambiae* using experimental houses built in a village and evaluate how such an effect compares with IN2CARE eave tubes.

## Methods

### Study area

The study was conducted in Kolongonouan village, near Bouaké city in central Côte d’Ivoire. The primary malaria vectors in the area are *Anopheles gambiae* sensu stricto and *An. coluzzii*, with widespread pyrethroid resistance driven by both target-site mutations and metabolic mechanisms [13]. Current vector control relies mainly on mass distribution of LLINs, with coverage exceeding 80% while IRS had never been implemented. Despite this, entomological surveillance reports moderate-to-high entomological inoculation rates (EIR), and malaria remains hyperendemic in Bouaké [14]. Houses in the village were built with modern materials (bricks, cement, and metal roofs) and only 20% had open eaves (Table 1). There were no screens on the windows or doors and, in all instances, houses visited were not entirely mosquito proof. A few gaps or slits were found beneath the door and across the window louvers even when these were closed in the day or during the night (Additional File 1: Supplementary Fig. S1).

**Table 1:** Number of houses and construction materials used in the study village.

Housing types	Number	Percentage (%)
Metal doors and windows	86	51.81
Wooden doors and windows	29	17.47
Mixed houses (wooden + metal or glass windows and doors)	51	30.72
Total	166	
Open eaves	35	21.08
Screens on the windows or doors	0	0.00

The types and materials used to build houses in the study village

### Experimental house

To simulate naturally poor house condition and study the impact that such condition may have on mosquito behavior, five identical houses were built next to each other in the village of Kolongonouan (7.674768−5.162976) on the basis of the predominant house type in the village. These houses described previously [14] had one bedroom, one living room, and a terrace (Additional File 2: Supplementary Fig. S2). The living room and bedroom had two windows (one at the front and one at the back side). House materials consisted of brick and cement, with metal roofs, wooden ceilings, metal doors, and windows with louvers. Windows and doors were also equipped with removable mosquito-proof screening (Additional File 3: Supplementary Fig. S3). When doors and windows were left open, panels of chicken wire that allowed natural airflow and mosquito entry and prevented room access by reptiles and rodents were used as replacement. Four holes to accommodate eave tubes were drilled per room (two on the front and two on the back side). As observed in the village houses [14], a 1-cm gap was left beneath the door as well as in louvers when these were closed (Additional File 4: Supplementary Fig. S4). An enclosure made of polyethylene netting, which could be opened and closed by zipper on all four sides of the houses was erected around each experimental house on the concrete base, covering the terrace entirely (Additional File 5: Supplementary Fig. S5). A sheet of plastic was used as roof over the entire house, to prevent the rain from entering the enclosure. A white plastic sheet was also placed on the floor of the enclosure to facilitate the collection of dead mosquitoes [14]. The door to the enclosure was placed on the front side of the house and closed with a zipper to prevent the escape of mosquitoes.

### Mosquito population.

*An. gambiae* used in the natural recruitment experiment were wild free-flying populations from the village of Kolongonouan, whereas those used in the release–recapture experiments were adult *An. gambiae* obtained from larvae collected within the city of Bouaké. Widespread pyrethroid resistance in *Anopheles gambiae* was recently documented in this area [13, 15], but no resistance update assays were conducted at the time of the study.

### Interventions and outcomes

The study assessed the efficacy of insecticide-treated screening (ITS) of windows alone or in combination with the IN2CARE eave tubes (ET) to protect households against malaria vectors.

The following combinations with these interventions were evaluated in the experimental houses between August and October 2022: (i) standard control house with small openings in the eaves by damaging one-fourth of the eave tube inserts, and no window screens (SCH); (ii) ITS of windows with damaged or open eaves as above (ITS op); (iii) ITS of windows + closed eaves with a piece of tarpaulin to prevent mosquito entry via eaves (ITS blq); (iv) ET alone without ITS (ET); and (v) SET (insecticide-treated eave tubes + untreated screening of windows (SET).

In the first instance, the impact of these treatments on indoor and outdoor mosquito density was assessed through natural recruitment. Release–recapture experiments in which large enclosures were built around individual houses and known number of mosquitoes released per night were then conducted to assess (i) the blood feeding and mortality of mosquitoes and (ii) the impact of holes in the ITS to simulate different levels of damage.

### Insecticide-treated materials

Eave tube inserts were treated with a 10% wettable powder formulation of β-cyfluthrin (Tempo 10 ^©^, Bayer) at 300–500 mg of powder per insert, already shown to be effective at killing pyrethroid-resistant mosquitoes [16, 17]. ITS was made from pieces of the roof material of PermaNet 3.0 (Vestergaard S.A., Switzerland) that contains PBO + deltamethrin. This product is known to be effective against pyrethroid-resistant mosquitoes in this area [18] and served as the “proof-of-concept” material.

### Experimental study procedure

#### Natural mosquito recruitment into the experimental houses

Community behavior in the study was slightly altered as described previously [14]. For example, windows were opened from 6:00 pm to 8:00 pm, then closed from 8:00 pm to 6:00 am, and re-opened from 6:00 am to 8:00 am. The front door was opened from 6:00 pm to midnight, closed from midnight to 5:00 am, and then re-opened from 5:00 am to 8:00 am. Doors and windows remained closed during daytime when there was no activity (i.e., from 8:00 am to 6:00 pm).

In each experimental house, one volunteer adult slept in the bedroom and another in the living room between 8:00 pm and 8:00 am under untreated mosquito nets. Two other volunteers were tasked to collect mosquitoes by human landing catches (HLC), one seating in the living room and the other one outdoor on the terrace. HLCs were conducted using hemolysis glass tubes and torch between 6:00 pm and 8:00 am, with replacement of volunteers halfway through by a second pair of volunteers. Supervisors were present to oversee the collection process and ensure that the doors and windows were closed or opened as appropriate. Treatments were rotated every 3 days between the experimental houses. This required 15 collection nights to complete one round of rotation. In total, two rounds were performed, that is, 30 collection nights, in a way that each treatment was evaluated six times in every house.

#### Release–recapture experiment to assess blood feeding and mosquito mortality

Each house had a sleeper in the bedroom and another in the living room under untreated net. A total of 100 non-blood-fed, 5-day-old female *An. gambiae*, starved for 6 hours without access to sugar meal, were released 15 min after sleepers entered their respective houses. At 5:00 am the following morning, sleepers collected mosquitoes within the house and the enclosure using torches and aspirators. The status (alive or dead, unfed or blood-fed) of the mosquitoes was recorded and survivors brought back to the laboratory then fed on a 10% sugar solution to monitor the mortality after 24 h holding. Sleepers rotated on consecutive nights, whereas treatments were evaluated on 3 nights in each house before rotating to the next. Thus, a total of 15 nights of release–recapture per treatment (i.e., one full rotation) and an average of 1500 mosquitoes were released.

For the ITS physical integrity release–recapture, the same ITS pieces were reused 2 months later, each deliberately holed with a 4 cm × 4 cm opening to simulate natural physical degradation [19]. Every eave was blocked with pieces of tarpaulin, while the doors and windows were closed and opened under the same condition as above.

Each treatment was evaluated over 3 nights in each house, with a total of 12 release–recapture nights per treatment (i.e., one full rotation) and 1200 mosquitoes released on average.

### Data analysis

Data were collected using Excel software, classified by experimental house and day of mosquito collection, then exported to STATA 18 for final analysis. The final dataset variables comprised treatment type, capture location, and key outcome parameters (blood-feeding and mortality rates). Linear regression was primarily used to assess the effect of the treatments on the number of females captured per location by performing pairwise comparisons between interventions. It was also used to evaluate the impact of the treatments on the mortality and blood-feeding rates, with weighting applied on the basis of the number of mosquitoes exposed, to account for differences in group sizes. This method allowed for a more accurate estimation of treatment effects while controlling for potential imbalances in data distribution. The analytical approach was applied to all treatment combinations to explore associations between interventions and outcome parameters.

## Results

### Impact on natural mosquito recruitment to house

**Fig. 1:**
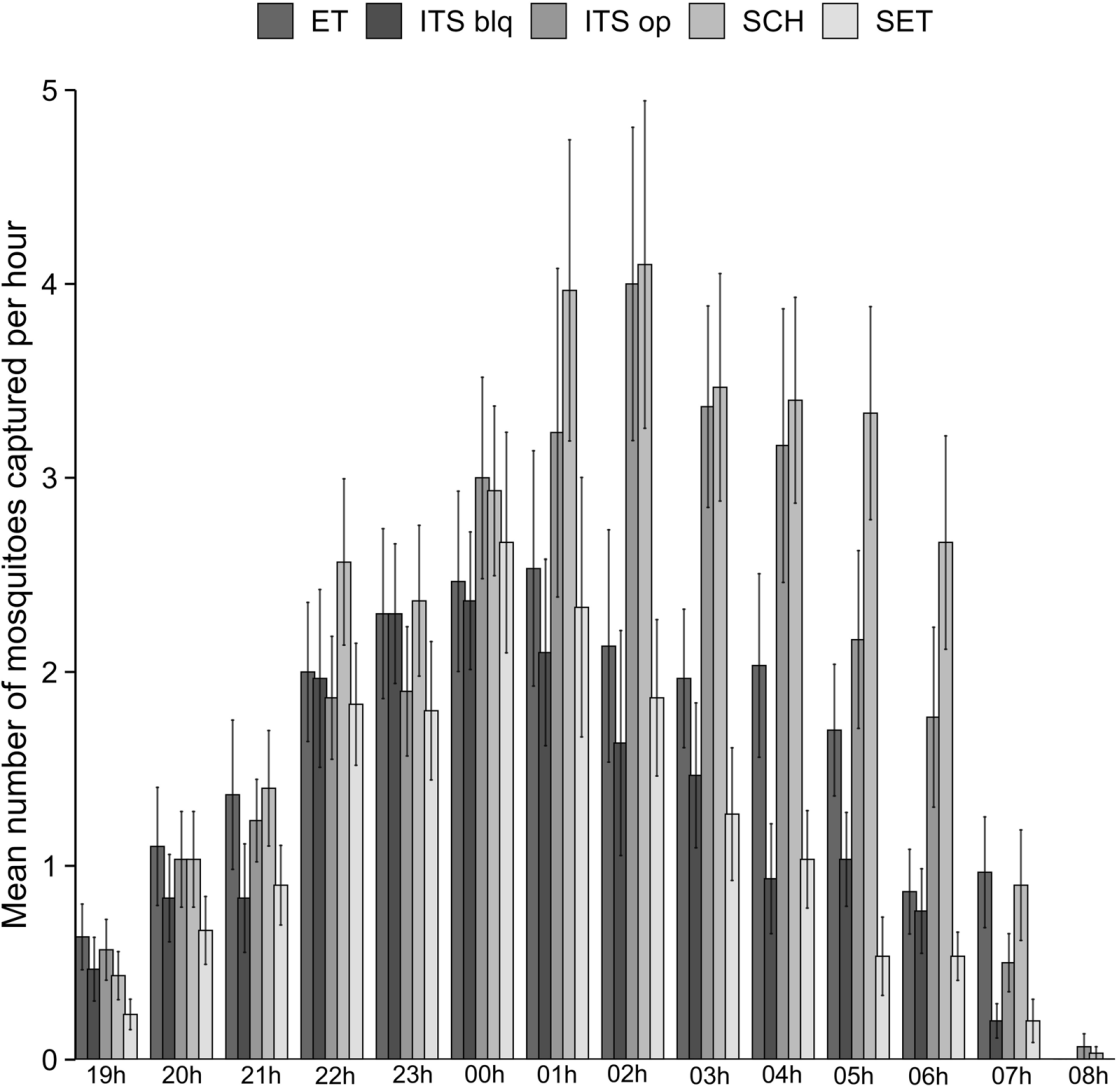
Mean number of *An. gambiae* captured per hour in experimental houses

All insecticide-treated materials significantly impacted *An. gambiae* house entry relative to the control SCH and ITS op (*P* < 0.01). ITS blq reduced *An. gambiae* entry rate by 31.8%, which was similar to reduction by ET (23.5%) (*P* = 0.1816) and SET (27.0%) (*P* = 0.9810). ITS op reduced by 18% the entry rate to house but the magnitude was not significant (*P* = 0.4019). SET had a greater impact on house entry prevention than ET alone (27% versus 23.5%; *P* = 0.0022) (Table 2). The peak recruitment period to houses happened between 11:00 pm and 3:00 am with on average between three and four *An. gambiae* accessing both the control SCH and ITS op houses per hour (Fig. 1). The outdoor mosquito collection session indicated no significant differences between any of the insecticide-treated material and the control SCH (*P* > 0.05).

**Table 2:** Natural recruitment. Density of *An. gambiae* (s.l.) inside and outside each experimental house

	SCH	ITS op	ITS blq	ET	SET
No. of females caught	1697	1389	1169	1299	1239
No. indoor	978^a^	836^a^	507^b^	662^c^	476^b^
Deterrence (%)	–	18.1	31.8	23.5	27.0
No. outdoor	719^a^	553^a, b, c^	662^a, b, c^	637^a, b, c^	763^a^

*SCH* standard untreated control house, *ITS op* insecticide-treated window screening with open eave, *ITS blq* insecticide-treated window screening with blocked eave, *ET* treated eave tubes without window screening, *SET* untreated window screening and treated eave tubes

Superscript letters a, b, and c indicate statistically significant differences between treatments within the same row (P 0.05; linear regression model). Values sharing the same letter do not differ significantly

### Impact of ITS on mosquito entry, blood feeding, and mortality in the release–recapture experiment

The trend for treatment impact on house entry in the release–recapture experiment within the enclosure mirrored that observed with the free flying recruitment within house.

None of the treatments protected against *An. gambiae* bites except the SET and ITS blq, which produced similar effects (blood-feeding rates: 26.8% [95% confidence interval [CI] 24.2–29.4] versus 25.9% [95% CI 23.4–28.5]; *P* = 0.8909) (Table 3).

**Table 3:** Summary of the release–recapture experiment with the resistant strain of *Anopheles gambiae* M'bé in experimental houses

	SCH	ITS op	ITS blq	ET	SET
Total recaptured	1361	1246	1197	1255	1136
No. within room	768	623	355	531	368
No. within enclosure	593	623	842	724	768
Blood fed (%)	40.1^a^	35.0^a^	25.9^b^	35.1^a^	26.8^b^
95% confidence limits	(37.50–42.77)	(32.34–37.71)	(23.43–28.47)	(32.49–37.85)	(24.20–29.43)
Blood feed inhibition (%)	**–**	12.7	35.7	12.5	33.4
24-h mortality (%)	6.2^a^	24.2^b^	33.7^c^	35.1^c^	40.7^c^
95% confidence limits	(4.95–7.58)	(21.88–26.72)	(30.99–36.42)	(32.41–37.77)	(37.80–43.59)

SCH, standard untreated Control House (with untreated eave tubes open 1/4); ITS_op, Insecticide Treated window Screening (with untreated eave tubes open 1/4); ET, Treated Eave Tubes; ITS_blq, Insecticide treated window screen with blocked untreated eaves tubes; SET, untreated window screen and treated eave tubes

Superscript letters a, b, and c indicate statistically significant differences between treatments within the same row (P 0.05; linear regression model). Values sharing the same letter do not differ significantly

All overnight mortality rates were significantly higher than that of the control SCH (*P* < 0.001) but similar between the ITS blq and ET (33.7% [95% CI 31.0–36.4] versus 35.1% [95% CI 32.4–37.8]; *P* > 0.05). SET induced the highest mortality rate (40.7% [95% CI 37.8–43.6]), though not significantly different from the level induced by ITS blq (*P* = 0.7076) or ET (*P* = 0.9653).

### Impact of holed ITS blq on house protection

Relative to the control, there was a significant reduction in blood feeding up to two holes in the intact ITS blq (47.4% inhibition rate; *P* < 0.05) but the effect disappeared after four holes (7.4% inhibition; *P* = 0.2665). There was no significant difference in mortality between two holes and four holes, although only the ITS blq with two holes induced mortality similar to that of the intact ITS blq (Table 4).

**Table 4:** Summary of the release–recapture experiment with the resistant strain of *Anopheles gambiae* from Bouaké in experimental houses

	UTS	ITS blq	ITS blq 2×	ITS blq 4×
Total recaptured	1125	914	1009	966
No. within room	419	227	280	327
No. within enclosure	706	687	729	639
Blood fed (%)	32.1^a^	25.9^b^	17.0^c^	29.9^a, b^
95% confidence limits	(29.3–34.9)	(23.11–28.90)	(14.77–19.51)	(27.04–32.91)
Blood feed inhibition (%)	**–**	19.8	47.4	7.4
24-h mortality (%)	3.2^a^	23.3^b^	18.23^b, c^	16.46^c^
95% confidence limits	(2.33–4.50)	(20.59–26.18)	(15.89–20.76)	(14.17–18.95)

*UTS* untreated window screening without holes, *ITS blq* insecticide treated window screening without holes, *ITS blq 2×* insecticide treated window screening with two holes, *ITS blq 4×* insecticide treated window screening with four holes

Superscript letters a, b, and c indicate statistically significant differences between treatments within the same row (P 0.05; linear regression model). Values sharing the same letter do not differ significantly

## Discussion

House-based interventions such as window screening are among the oldest methods against vector-borne diseases [20]*.* However, their impact on malaria transmission and factors affecting their effectiveness remain unclear. The aim of this study was to assess at household level within a village, the entomological impact of insecticide-treated screening (ITS) of windows on the behavior of insecticide resistant *An. gambiae* and how such effect compared with IN2CARE eave tubes.

The study demonstrated a significant reduction in indoor mosquito density within houses equipped with the ITS and eaves of the house closed (ITS blq) compared with the standard control house with small openings at eave level (SCH). Similar reductions were observed with houses equipped with eave tubes alone (ET) or SET (insecticide-treated eave tubes + untreated screening of windows). In the release–recapture experiment, ITS blq, SET, and ET were equally effective in reducing mosquito entry. Screening houses to prevent mosquito entry has proven effective in different settings. For example, a study in Ethiopia showed that screening doors and windows and closing eaves with mud reduced indoor *An. arabiensis* density by 40% [21]. Similarly, a study in The Gambia found that screening windows and doors plus closing eaves reduced indoor density by 59% [10]. Other studies reported higher malaria vector density in poorly constructed houses compared with improved structures [22–24].

In the current study, optimum protection against mosquito bites was reached after midnight when most residents had closed their doors and gone to bed. These findings under real-life condition highlight the influence of human behavior on the effectiveness of malaria control interventions, which may differ from results obtained under controlled experimental conditions. For example, experimental hut trials evaluating product efficacy often do not account for human behavior, such as residents staying awake late at night with doors or windows open during dry and hot seasons [14]. Further research is needed to assess the impact of opened and closed doors/windows at different time points from the evening on the effectiveness of vector control tools. Under semi-field condition, using experimental houses, ITS and eave tubes substantially reduced indoor mosquito entry but not outdoor density. These results must be interpreted cautiously as the current study measured individual protection and potential of the intervention to reduce malaria transmission. The results could be different if these tools were evaluated at community level where they could produce mass killing effect of mosquitoes and potentially reduce outdoor density and transmission of malaria. In addition to reducing indoor densities of *An. gambiae*, ITS of windows also impacted mosquito mortality and blood-feeding rates against pyrethroid-resistant populations. Results from the second experiment showed higher mosquito mortality in all the ITS-treated houses relative to control. Only the ITS blq and SET reduced in similar proportion blood-feeding rates of *An. gambiae*. The efficacy of SET in decreasing the blood-feeding rates and increasing the mosquito mortality has been previously reported in malaria vectors [14, 17]. Interestingly, ITS blq was as effective as SET in preventing mosquito blood feeding while maintaining insecticidal effects. Similar findings were reported in Mexico, where ITS installation reduced mosquito blood-feeding rates, even a year later [25–27]. Randomized controlled trials also showed ITS to reduce prevalence of anemia in children, suggesting its impact on the mosquito blood feeding and mortality in affected regions [10]. With potential for preventive measure, ITS of windows with long-lasting product and material may provide long-term benefits to households, as it is less invasive and easy to implement strategy compared with eave tubes which involves drilling of homes in addition to maintenance. High levels of satisfaction and acceptance of ITS as a protective method against mosquito bites have been observed in Mexico [27]. However, operational challenges, such as physical degradation of the material in the ITS as well as resistance of the active ingredients (AIs) to ultraviolet (UV) light may affect its durability in the field. No insecticide resistance testing was performed during the study period; However, we relied on a recently published data describing the resistance status in *An. gambiae* within the same area [15]. As the resistance profile may change over time, reliance on prior data is a limitation that should be considered when interpreting our findings.

The intensive use of long-lasting insecticidal nets (LLINs) in the field has often been associated with a decline in their long-term effectiveness. In this study, the top surface of a new-generation LLIN (PermaNet 3.0 LN, containing deltamethrin and piperonyl butoxide) designed to control resistance mediated by oxidases was used as the screening material in the ITS, while the eave tube inserts were treated with a pyrethroid (β-cyfluthrin). Previous laboratory and semi-field studies indicated that electrostatic coating of pyrethroid powder on inserts was highly effective against mosquitoes despite pyrethroid resistance in them [7]. Furthermore, clinical trial of eave tubes treated with β-cyfluthrin reduced the incidence of malaria by nearly 40% in the area adjacent to our current trial area where the malaria vector exhibited a similarly high level of insecticide resistance [5].

It is important to note that the aim was to provide a proof of concept and not to advocate for repurposing LLINs in this way. Hopefully the encouraging results will provide motivation for further product development research to develop robust screening materials with appropriate, easy to use fitting and fastening. A related study demonstrated that PermaNet 3.0 LN used in eave tubes achieved over 50% mortality in pyrethroid-resistant *An. gambiae*, reinforcing the potential of LLIN-derived materials in lethal house-lure strategies [28]. However, their study also highlighted rapid loss of efficacy under environmental exposure, and our own findings showed that holes in the screening material significantly compromised its protective effect. These insights suggest that future ITS development should prioritize purpose-designed, weather-resistant materials with sustained insecticidal activity and improved physical durability. With the widespread increase in pyrethroid resistance across Sub-Saharan Africa, there is impetus to develop new insecticides with distinct mode of action such as chlorfenapyr, clothianidin, or broflanilide for use in ITS and eave. Considering that the SET demonstrated significant epidemiological impact in areas with pyrethroid-resistant *An. gambiae*, scaling up ITS could yield similar effects, although implementation of ITS versus eave tubes at scale may come at different costs. Future studies must comprise cost-effectiveness analysis to assess cost and feasibility at scale. This must include existing tools, such as LLINs, to optimize malaria prevention strategies.

## Conclusions

This study demonstrates the potential of insecticide-treated screening (ITS) to protect households and reduce malaria transmission in areas highly affected by insecticide resistance. ITS showed promise as an effective tool for reducing the mosquito entry and the blood feeding while killing mosquitoes that attempted to enter households. However, operational challenges such as physical and insecticidal degradation in the screen and variation in household behavior could compromise its long-term effectiveness. Studies comparing product efficacy under real life conditions in controlled houses with human behavior involvement and in experimental huts must be conducted to assess the differences in impact on the mosquito mortality and blood feeding, which are key indicators of malaria transmission.

## Supplementary Information

Additional file 1: Figure S1: Example of house not entirely mosquito proof in the study village.

Additional file 2: Figure S2: Plan of experimental houses.

Additional file 3: Figure S3: Experimental houses equipped with removable mosquito-proof screening used for natural recruitment experiment.

Additional file 4: Figure S4: 1 cm slits under the louvers of closed doors and windows simulating the living conditions of rural households.

Additional file 5: Figure S5: Experimental house with retracted (**a**) and closed (**b**) enclosure.

## Data Availability

Data supporting the main conclusions of this study are included in the manuscript.

## Abbreviations

AIs: Active ingredients
LLINs: Long-last insecticide nets
IRS: Indoor residual spraying
ITNs: Indoor residual spraying (IRS) and insecticide-treated nets
ITS: Insecticide-treated screening
ITS op: ITS of windows with small openings in the eaves
ITS blq: ITS of windows with eaves blocked to prevent mosquito entry via eaves
ET: Insecticide treatment applied to the eaves using In2Care eave tubes, without window screening
SCH: Standard control house with small openings in the eaves and no window screens
SET: Untreated window screening together with insecticide-treated eave tubes
