# Supplementary figures and images for: Insecticide-treated screening of windows for household protection against insecticide-resistant Anopheles gambiae sensu lato in Côte d’Ivoire: a semi-field trial
Source: Parasit Vectors. 2025 Dec 30;19:60. doi: 10.1186/s13071-025-07194-z (PMC12859916; doi:10.1186/s13071-025-07194-z)

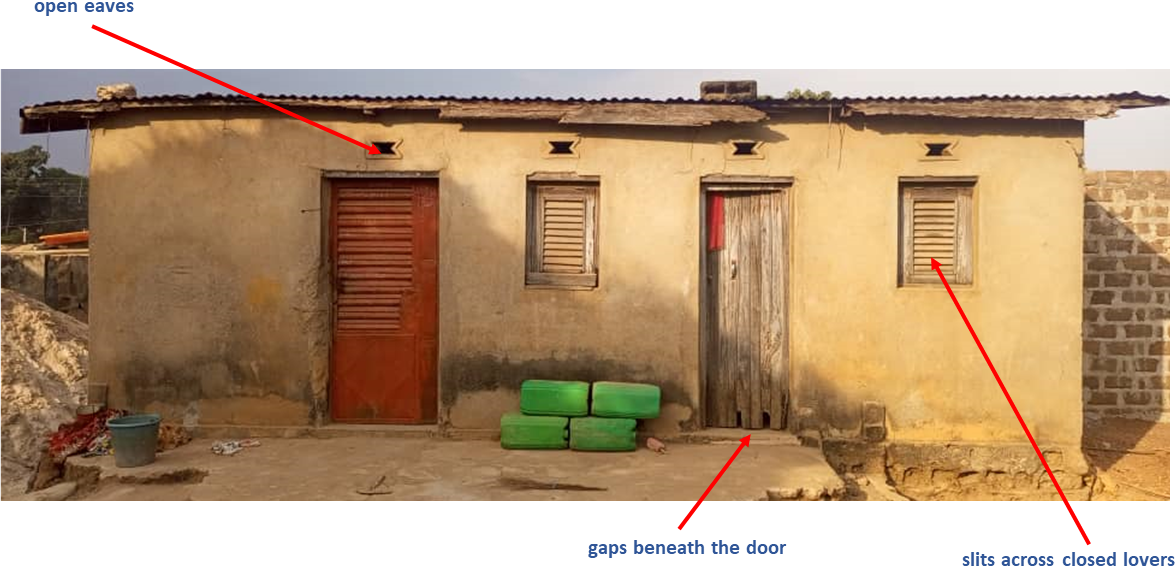

Supplement: Supplementary file 1 — Additional file 1: Figure S1: Example of house not entirely mosquito proof in the study village. [file 13071_2025_7194_MOESM1_ESM.tif]

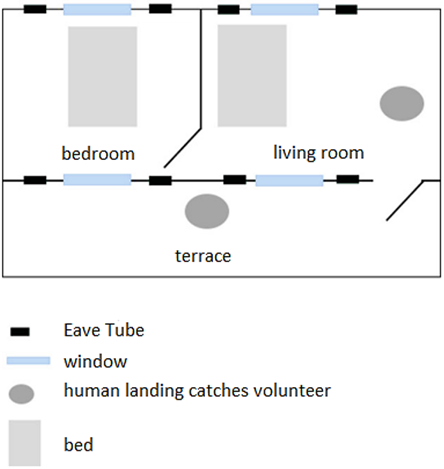

Supplement: Supplementary file 2 — Additional file 2: Figure S2: Plan of experimental houses. [file 13071_2025_7194_MOESM2_ESM.tif]

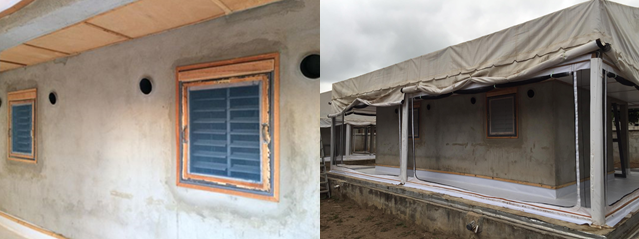

Supplement: Supplementary file 3 — Additional file 3: Figure S3: Experimental houses equipped with removable mosquito-proof screening used for natural recruitment experiment. [file 13071_2025_7194_MOESM3_ESM.tif]

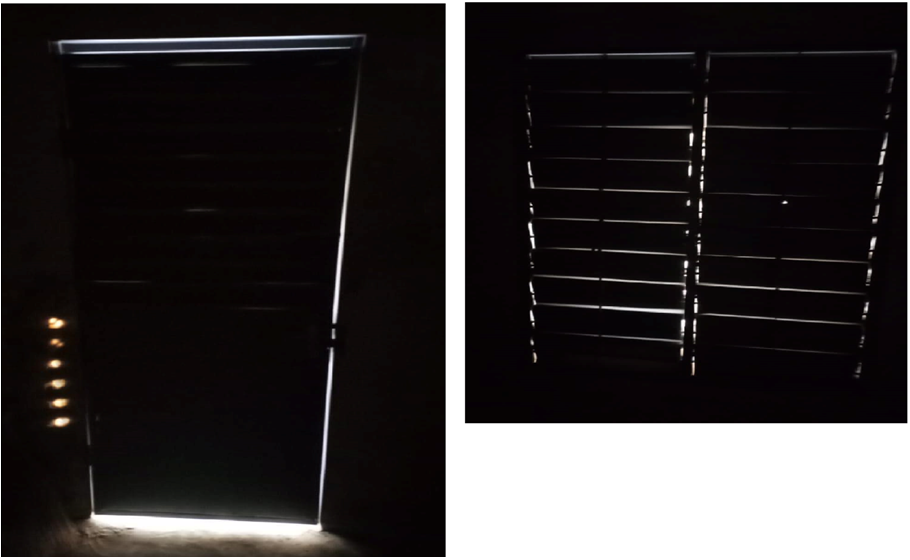

Supplement: Supplementary file 4 — Additional file 4: Figure S4: 1 cm slits under the louvers of closed doors and windows simulating the living conditions of rural households. [file 13071_2025_7194_MOESM4_ESM.tif]

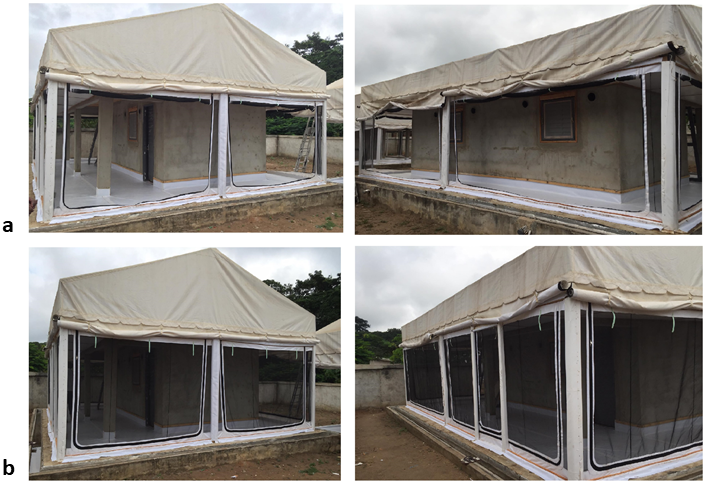

Supplement: Supplementary file 5 — Additional file 5: Figure S5: Experimental house with retracted (a) and closed (b) enclosure. [file 13071_2025_7194_MOESM5_ESM.tif]
